# CD36 Shunts Eicosanoid Metabolism to Repress CD14 Licensed Interleukin-1β Release and Inflammation

**DOI:** 10.3389/fimmu.2018.00890

**Published:** 2018-04-27

**Authors:** Karina F. Zoccal, Luiz G. Gardinassi, Carlos A. Sorgi, Alyne F. G. Meirelles, Karla C. F. Bordon, Isaias Glezer, Palmira Cupo, Alessandra K. Matsuno, Valdes R. Bollela, Eliane C. Arantes, Francisco S. Guimarães, Lúcia Helena Faccioli

**Affiliations:** ^1^Departamento de Análises Clínicas, Toxicológicas e Bromatológicas, Faculdade de Ciências Farmacêuticas de Ribeirão Preto, Universidade de São Paulo, Ribeirão Preto, Brazil; ^2^Departamento de Física e Química, Faculdade de Ciências Farmacêuticas de Ribeirão Preto, Universidade de São Paulo, Ribeirão Preto, Brazil; ^3^Departamento de Bioquímica, Escola Paulista de Medicina, Universidade Federal de São Paulo, São Paulo, Brazil; ^4^Departamento de Puericultura e Pediatria, Faculdade de Medicina de Ribeirão Preto, Universidade de São Paulo, Ribeirão Preto, Brazil; ^5^Departamento de Clínica Médica, Faculdade de Medicina de Ribeirão Preto, Universidade de São Paulo, Ribeirão Preto, Brazil; ^6^Departamento de Farmacologia, Faculdade de Medicina de Ribeirão Preto, Universidade de São Paulo, Ribeirão Preto, Brazil

**Keywords:** interleukin-1β, leukotriene B_4_, prostaglandin E_2_, cyclic adenosine monophosphate, CD36 receptor, CD14 receptor, venom

## Abstract

Interleukin (IL)-1β is a potential target for treatment of several inflammatory diseases, including envenomation by the scorpion *Tityus serrulatus*. In this context, bioactive lipids such as prostaglandin (PG)E_2_ and leukotriene (LT)B_4_ modulate the production of IL-1β by innate immune cells. Pattern recognition receptors (PRRs) that perceive *T. serrulatus* venom (TsV), and orchestrate LTB_4_, PGE_2_, and cyclic adenosine monophosphate (cAMP) production to regulate IL-1β release are unknown. Furthermore, molecular mechanisms driving human cell responses to TsV remain uncharacterized. Here, we identified that both CD14 and CD36 control the synthesis of bioactive lipids, inflammatory cytokines, and mortality mediated by TsV. CD14 induces PGE_2_/cAMP/IL-1β release and inflammation. By contrast, CD36 shunts eicosanoid metabolism toward production of LTB_4_, which represses the PGE_2_/cAMP/IL-1β axis and mortality. Of importance, the molecular mechanisms observed in mice strongly correlate with those of human cell responses to TsV. Overall, this study provides major insights into molecular mechanisms connecting CD14 and CD36 with differential eicosanoid metabolism and inflammation mediated by IL-1β.

## Introduction

Inflammation is a critical process for the host response against pathogens and other harmful stimuli, such as toxins. However, fine-tuned regulatory mechanisms evolved to avoid adverse effects from excessive inflammation (1, 2). Innate immune cells, including macrophages, drive these responses by secreting a wide spectrum of soluble mediators (1, 3). Among them, bioactive lipids play critical roles to initiate and resolve inflammation (4). Activated innate immune cells produce and secrete eicosanoids, bioactive lipids derived from the ω-6 polyunsaturated fatty acid, arachidonic acid (AA). Prostaglandin (PG) synthesis begins with AA oxygenation by cyclooxygenases 1 and 2 (COX1/2), while leukotrienes (LTs) derive from 5-lypoxygenase pathway activity (5-LO) (5). PGs and LTs mediate pleiotropic functions in inflammation, such as amplification or regulation of cytokine release (3, 6) and recruitment of leukocytes (7–9). These events are triggered after pattern recognition receptors (PRRs) engage ligands of diverse chemical nature (1, 2, 10, 11). Toll-like receptors (TLRs) compose a well-characterized family of PRRs, which recognize microbial products, endogenous signals of cellular damage, as well as toxins and venom compounds (2, 10–12). TLRs function cooperatively with each other or with several co-receptors, such as CD14 and CD36, to activate a variety of inflammatory signaling cascades (13–17).

Recently, we observed that mouse macrophages recognize *Tityus serrulatus* venom (TsV) through TLRs to mediate the production of inflammatory cytokines and bioactive lipids (11). Moreover, we demonstrated that TsV induces NLRP3 inflammasome assembly, caspase-1 activation, and interleukin (IL)-1β maturation, which culminates in lung edema and death of envenomed mice (3). Inflammasome/caspase-1 activation and subsequent IL-1β release is potentiated by mechanisms that induce PGE_2_/cyclic adenosine monophosphate (cAMP) production, but repressed by LTB_4_ (3, 18, 19). In humans, several inflammatory cytokines are released in the circulation following a scorpion sting (20–22). While these studies revealed important immunopathological features of scorpion envenomation, PRRs that perceive effector TsV compounds, and induce bioactive eicosanoids to regulate IL-1β release remain unknown. In addition, it is still unclear whether the same mechanisms translate into inflammatory responses during scorpion envenomation in humans.

Both CD14 and CD36 display fundamental roles not only as co-receptors but also as major regulators of the inflammatory response (17). Their functions are dictated by TLR-dependent and -independent signaling cascades (23–25). CD14 is a glycoprotein abundantly expressed in membranes of myeloid cells and commonly used as a monocyte/macrophage marker (26). Upon activation, monocytes secrete soluble form of CD14 (sCD14) (27), whereby several inflammatory conditions are associated with increased levels of circulating sCD14 (27–29). Widely studied due its role as a lipopolysaccharide (LPS) receptor, CD14 is also required for maximum production of tumor necrosis factor (TNF)-α, IL-6, and PGE_2_ by TsV-stimulated mouse macrophages (11). CD36 is expressed by multiple cell types and tissues, and binds to several pathogen and endogenous ligands (30, 31). Belonging to the class B scavenger receptor family, it has significant roles in angiogenesis and control of fatty acid uptake (30). In addition, CD36 has emerged as a central molecule for inflammasome activation and consequent secretion of IL-1β during sterile inflammation (16).

Here, we show that CD14 induces a robust inflammatory response to TsV, controlling the production of PGE_2_, cAMP, and IL-1β by mouse macrophages *in vitro*, while absence of CD14 reduces lung inflammation and edema, increasing survival after envenomation *in vivo*. Strikingly, we identified a surprising role of CD36 on the regulation of TsV-induced IL-1β release. CD36 specifically represses IL-1β secretion by driving eicosanoid metabolism toward LTB_4_ synthesis. Indeed, a CD36-deficient mouse strain (*Cd36^obl/obl^*) is highly susceptible to envenomation, while treatment with LTB_4_ rescues mice from fatal outcomes. Of importance, we confirmed that PGE_2_ levels are correlated with and boosts IL-1β production by human peripheral blood mononuclear cells (PBMCs) stimulated with TsV. However, differential activity of enzymes involved in eicosanoid metabolism coordinates IL-1β levels.

## Materials and Methods

### Healthy Human Subjects and Envenomed Patients

Peripheral blood (40 mL), peripheral blood mononuclear cells (PBMCs) and plasma samples were collected using tubes with heparin (BD Biosciences, San Diego, CA, USA) from healthy subjects of both sexes (*n* = 24) who had not taken anti-inflammatory drugs, had not used anticoagulant, or had not chronic inflammatory and infectious diseases (see Tables S1 and S2 in Supplementary Material). These procedures were approved by the School of Pharmaceutical Sciences of Ribeirão Preto Human Research Ethics Committee (protocol #54426115.1.0000.5403) and all subjects provided written informed consent.

Plasma samples were also collected from patients envenomed by *T. serrulatus* before treatment (*n* = 14, women to men ratio = 1.8, mean age ± SD = 5.6 ± 7.3 years). These procedures were approved by the Clinics Hospital of Ribeirão Preto Medical School Research Ethics Committee (protocol #54426115.1.3001.5440) and all patients provided written informed consent.

### Whole Blood Flow Cytometry Analysis

Aliquots of fresh circulating blood (containing 1 × 10^6^ leukocytes) were collected from 10 healthy subjects. Samples were incubated with vehicle or TsV for 4 h, and cell surface expression of CD14 and CD36 was determined by flow cytometry using fluorochrome-conjugated antibodies (BD Biosciences, NJ, USA). Whole blood cells were stained with fluorescein isothiocyanate-conjugated anti-CD36 and allophycocyanin-conjugated anti-CD14 antibodies. The cells were then washed with PBS containing 2% fetal bovine serum, pelleted by centrifugation at 400 × *g* and fixed with PBS containing 1% (w/V) paraformaldehyde. Data were acquired using a FACSCanto flow cytometer (Becton Dickinson, CA, USA) and analyzed with FlowJo software (Tree Star).

### Peripheral Blood Mononuclear Cells (PBMCs) Isolation, TsV Stimulation, and Pharmacological Treatments

PBMCs were isolated using Ficoll-Paque PLUS (GE Healthcare, Uppsala Sweden) density gradient centrifugation following the manufacturer’s instructions. PBMCs were plated at the density of 1 × 10^6^ cells/well in 300 µL serum-free RPMI supplemented with antibiotics (incomplete RPMI-1640). The adhered cells were then cultured at 37°C (5% CO_2_) for 2 h. Next, the cells were pre-incubated with or without indomethacin (30 min; 10 µM; Cayman Chemical, MI, USA), MK886 (30 min; 10 µM), PGE_2_ (10 min; 10 µM; Cayman Chemical), LTB_4_ (10 min; 100 nM; Cayman Chemical), or dexamethasone (1 h; 0.1 µM; Aché Laboratórios, São Paulo, Brazil). PGE_2_ and LTB_4_ from ethanol stock solution were suspended in serum-free RPMI, and an equivalent volume of ethanol (not more than 0.01%) was added to the RPMI; this solution was designated as the vehicle control. Indomethacin, MK886 (prediluted in PBS/0.01% ethanol), and dexamethasone were diluted in serum-free RPMI. RPMI containing the same proportion of ethanol was used to dilute the compounds, yielding the vehicle control. After treatment, PBMCs were stimulated with TsV (50 µg/mL) for 24 h at 37°C in a humidified atmosphere with 5% CO_2_. Subsequently, the supernatants were collected for IL-1β quantification, and the cell lysate was used for analysis of gene expression.

### Mice

This study was carried out in accordance with the institutional guidelines for ethics in animal experiments approved by the Animal Care Committee of the Campus of Ribeirão Preto (PCARP) at the University of São Paulo, Ribeirão Preto, Brazil (protocol #14.1.272.53.7). Six- to eight-week-old female or male mice were used for *in vivo* and *in vitro* experiments. The mice were matched by sex and age for all procedures. *Cd14*^−/−^ (32), *Tlr2*^−/−^ (33), and *Tlr4*^−/−^ (34) mice were backcrossed with C57BL/6 mice and were bred and maintained at the animal facilities of the Ribeirão Preto Medical School, University of São Paulo. *Cd36^obl^*/Mmucd mice (15) were acquired from MMRRC (UC Davis) in heterozygosis and were inter-crossed to yield homozygotes mutants and wild-type mice; genotyping was performed by PCR followed by DNA sequencing as described (35). C57BL/6 (wild type) mice were obtained from the animal facilities of the PCARP, at the University of São Paulo, Ribeirão Preto, Brazil. Sample size was determined based on previous studies from our laboratory and literature and considering alpha and beta errors of 0.05 and 0.20, respectively. We observed TsV-inoculated mice for 8 h. The time of death was used for survival analysis. The surviving mice were euthanized with an overdose of chemical anesthetics (100 mg/kg ketamine and 10 mg/kg xylazine) at 8 h after envenomation or vehicle inoculation.

### TsV, Ts1, and the Fatty Acid-Rich Fraction

*Tityus serrulatus* scorpions were maintained at the vivarium of the Ribeirão Preto Medical School, University of São Paulo, in accordance with the Brazilian Institute of Environment. TsV was obtained by electrical stimulation, and Ts1 toxin (representing 16% of the total crude soluble TsV) was obtained as described previously (36). TsV and purified Ts1 were stored at −20°C until use. Prior to the experiments, TsV and Ts1 were diluted in PBS and filtered through a 0.22-µm sterilizing membrane (Millipore, Tullagreen, CO, USA). *Limulus* Amebocyte Lysate tests (QCL-1000; Bio Whittaker, Cambrex Company, Walkersville, MD, USA) were performed to detect LPS in the TsV and Ts1 samples, according to the manufacturer’s instructions. No LPS was detected in TsV and Ts1 samples. To prepare the fatty acid-rich fraction, desiccated TsV (5 mg) was suspended in 200 µL cold ultrapure water (18.2 MΩ cm; Milli-Q water; Millipore, Bedford, MA, USA) and centrifuged at 12,400 × *g* and 4°C for 10 min. The supernatant was separated, and the precipitate was washed twice with 100 µL cold ultrapure water and centrifuged as described above. This precipitate containing mucus and fatty acids was designated the fatty acid-rich fraction, and the presence of lipids was confirmed by mass spectrometry.

### Activation of Mouse Peritoneal Macrophages by TsV and *In Vitro* Pharmacological Treatments

Resident peritoneal macrophages from naïve C57BL/6, *Cd14*^−/−^, *Cd36^obl/obl^, Tlr2*^−/−^, and *Tlr4*^−/−^ mice were harvested by peritoneal washes with RPMI-1640 and were plated at a density of 2 × 10^5^ cells/well in 200 µL serum-free RPMI supplemented with antibiotics (incomplete RPMI-1640). The cells were then cultured at 37°C (5% CO_2_) for 2 h. Next, the supernatants were removed, and the cells were pre-treated with or without *Escherichia coli* 01112.B4 LPS (1,000 ng/mL; Sigma-Aldrich, St. Louis, MO, USA) for 4 h. Next, PBS (vehicle), TsV, the fatty acid-rich fraction, or Ts1 (50 µg/mL) in 200 µL incomplete RPMI was added, and the cells were incubated at 37°C in a humidified atmosphere containing 5% CO_2_. After 24 h, the supernatants were collected for IL-1β, PGE_2_, LTB_4_, IL-6, and TNFα quantification. In separate experiments, 1 × 10^6^ peritoneal macrophages/well in 48-well plates, collected from C57BL/6, *Cd36^obl/obl^*, and *Cd14*^−/−^ mice, were incubated with incomplete RPMI medium following stimulation with or without TsV (50 µg/mL) for 4 or 24 h at 37°C in a humidified atmosphere containing 5% CO_2_ for analysis of mRNA expression by quantitative real-time reverse transcription polymerase chain reaction (qRT-PCR). When indicated and before TsV stimulation, the cultured mouse peritoneal macrophages were pre-treated with forskolin (an adenylate cyclase agonist, 10 or 25 µM; 10 min; Sigma-Aldrich), MK886 (a FLAP inhibitor, 10 µM; 30 min), indomethacin (a COX1/2 inhibitor; 10 µM; 30 min, Cayman Chemical), H89 dihydrochloride hydrate [a protein kinase A (PKA) inhibitor, 25 µM, 2 h; Sigma-Aldrich], LTB_4_ (100 nM; 10 min; Cayman Chemical), or U75302 (a BLT1 antagonist, 1 or 10 µM; 30 min; Cayman Chemical). The same volume of the solvent used to suspend the compounds was added to RPMI (as the vehicle). Culture conditions, time, and concentration of each compound were based on our previous publication (3).

### Quantitative Real-Time Reverse Transcription Polymerase Chain Reaction

RNA was extracted using a guanidine-based column method, according to the manufacturer’s protocol (Purelink, Ambion), and the quantity of RNA was determined by means of a fluorometric assay (Qbit; Invitrogen, Carlsbad, CA, USA). cDNA was synthesized from 1 µg total RNA (High Quality cDNA Reverse Transcriptase Kits; Applied Biosystems, Foster City, CA, USA). Aliquots (2 µL) of total cDNA were amplified by qRT-PCR using TaqMan primers for human: *PTGS2, CD14, CD36, TLR2, TLR4, ALOX5AP, ALOX-5*, and *CASP1* or mice: *Alox5, Alox5ap, Cd14, Cd36, Casp1, Tlr2i*, and *Tlr4* in a StepOne Plus machine (Applied Biosystems). *Gapdh* and *Actb* were used as reference genes. Reactions were performed in duplicate, and amplification was performed under the following conditions: denaturation at 95°C for 2 min, followed by 40 cycles of 95°C for 2 s and 60°C for 20 s. The results were normalized to the expression levels of the endogenous internal controls *Actb* and *Gapdh*. The ΔΔCt method was used for the analysis.

### *In Vivo* Experiments and Pharmacological Treatments

In all experiments under all conditions, mice were weighed before experiments. *Cd36^obl/obl^, Cd14^−/−^*, and C57BL/6 mice without treatment were inoculated with a lethal dose of TsV or PBS (vehicle) (3). C57BL/6 or *Cd36^obl/obl^* mice were treated with or without 20 µL of LTB_4_ (Cayman Chemical—50 ng/mouse) *via* i.n. administration, or 20 µL of vehicle (PBS + 0.05% ethanol). LTB_4_ or vehicle was administered 2 and 0.5 h before the lethal dose of TsV (180 µg/kg). In an additional experiment, C57BL/6 or *Cd14^−/−^* mice were treated with or without BLT1 antagonist (U75302, 50 ng/mouse, i.n.) in 20 µL vehicle (Cayman Chemical). BLT1 antagonist or vehicle (PBS + 0.05% ethanol) was administered 24, 12, and 2 h before inoculation with a lethal dose of TsV (180 µg/kg). Animals inoculated with 200 µL PBS (i.p.) were used as negative controls. Lungs were excised immediately after euthanasia (overdose of 20% ketamine plus 10% xylazine) 8 h after the injection of TsV or vehicle. Lungs were weighed, and 200 mg of tissue was homogenized in 2 mL incomplete RPMI. After centrifugation (400 × *g* for 10 min), the supernatants were transferred to new tubes, split into two samples of 1 mL each, and stored at −80°C until use. For measurement of myeloperoxidase (MPO) activity, one lobule of a lung was cut, instantly frozen in liquid nitrogen, and stored at −80°C until use. Weighed lungs were used for the calculation of the lung/body weight index to evaluate lung edema. Samples of lung parenchyma were processed and stained with hematoxylin and eosin for histological analysis by blinded observers. Analysis of these sections was performed with a video camera (Leica Microsystems Ltd., Heebrugg, Switzerland) connected to a Leica microscope DMR (Leica, Microsystems GmbH, Wetzlar, Germany) attached to a computer. Images were processed by Leica QWin software (Leica Microsystems Image Solutions, Cambridge, UK).

### Quantification of Soluble Mediators and MPO

Interleukin-1β, IL-6, and TNF-α present in the supernatants of lung homogenates or cell cultures were quantified using enzyme-linked immunosorbent assay (ELISA) kits (R&D Systems, Minneapolis, MN, USA). Lipids were purified from 1 mL of filtered supernatants from the lung homogenates using Sep-Pak C_18_ cartridges (Thermo Fisher Scientific, Bellefonte, PA, USA). Measurements of LTB_4_ and PGE_2_ concentrations of lungs or cell cultures were performed by enzyme immunoassays (Enzo Life Sciences, NY, USA). Supernatants of the lung homogenates were used for measurement of MPO activity as described previously (37). Total protein was quantified using Coomassie Protein Assay Reagent (Pierce Chemical, Rockford, IL, USA). Levels of human soluble CD14 (sCD14) were determined by Quantikine Human sCD14 Immunoassay (R&D Systems Inc., Minneapolis, MN, USA). Levels of PGE_2_ secreted by human PBMCs were measured by LC-MS/MS as described previously (38, 39).

### Quantification of Intracellular cAMP

For intracellular cAMP measurement, peritoneal macrophages were harvested from naïve C57BL/6, *Cd36^obl/obl^*, or *Cd14*^−/−^ mice, and 1 × 10^6^ cells/well were seeded in 48-well plates. The plates were then incubated with 1 mL serum-free RPMI with or without TsV (50 µg/mL) for 5 min at 37°C in 5% CO_2_. Culture supernatants were aspirated, and the cells were lysed by incubation for 10 min with 0.1 M HCl at room temperature, followed by disruption using a cell scraper (3). Intracellular cAMP was quantified by ELISA, according to the manufacturer’s acetylation protocol (Enzo Life Sciences, Farmingdale, NY, USA).

### Statistical Analyses

For comparison of multiple groups of animals, we performed one-way analysis of variance (ANOVA) or Kruskal–Wallis tests, followed by Bonferroni’s or Dunn’s multiple comparison tests, respectively. Differences between two groups were evaluated using paired or unpaired two-tailed Student’s *t*-tests. Shapiro–Wilk normality test was used to evaluate data distribution and gene expression was evaluated using one-sample *t*-test or Wilcoxon signed rank test. Differences in survival were analyzed using log-rank tests. Pearson’s correlation was used to identify significant associations. All calculations were performed in GraphPad Prism 5.0 software (GraphPad, San Diego, CA, USA). Differences with *P* values of less than 0.05 were considered statistically significant.

The frequency of high cytokine producers was calculated as previously proposed by Luiza-Silva et al. (40). Briefly, the global median cytokine frequency was calculated for each PBMC subpopulation using the entire data universe (*n* = 24 subjects), each subject was categorized as a “low” (below the global median) or “high” (higher than or equal to the global median) responder.

## Results

### Opposing Roles of CD14 or CD36 on the Induction of IL-1β by TsV-Stimulated Mouse Macrophages

**Figure 1:**
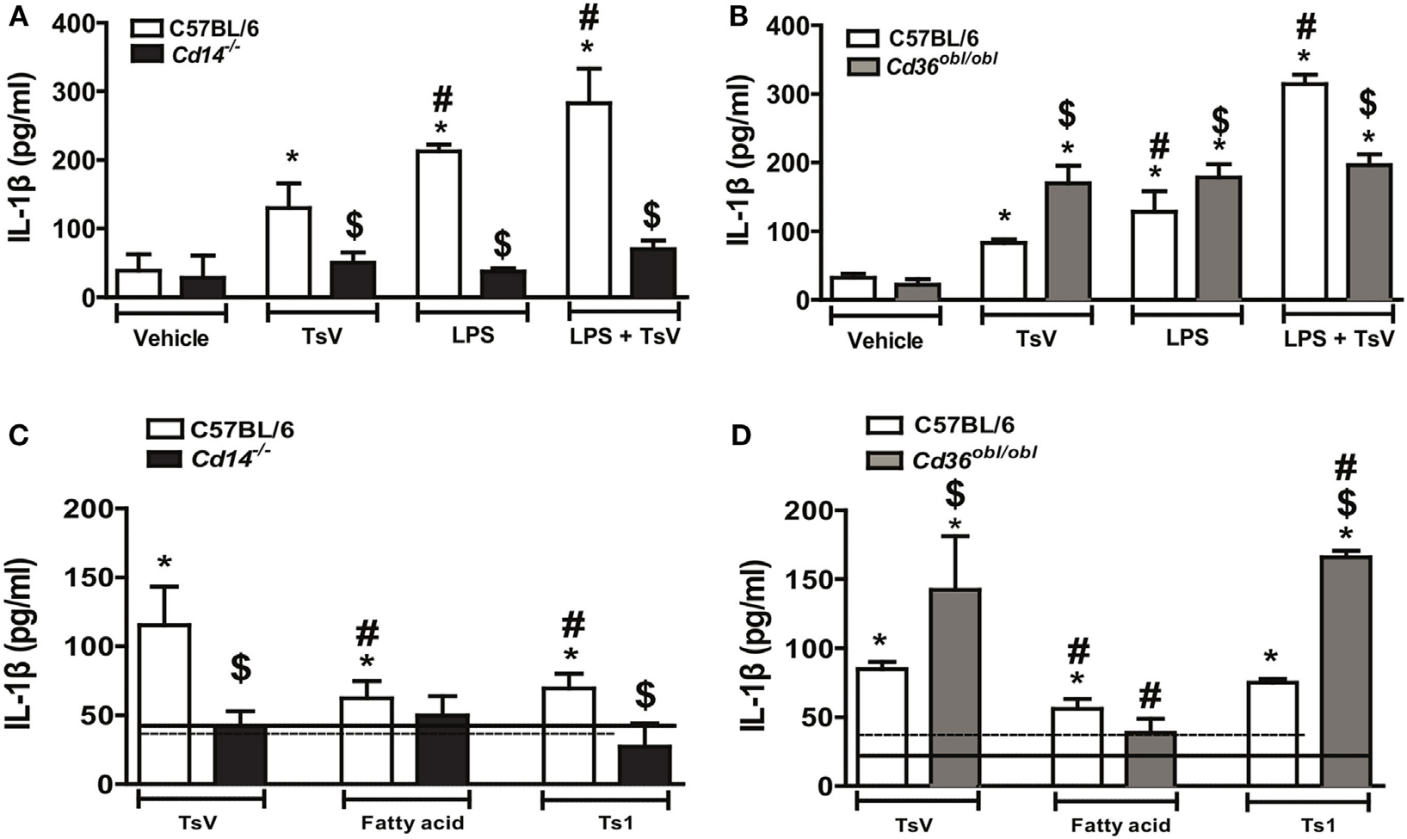
Differential regulation of interleukin (IL)-1β promoted by CD14 and CD36 is independent of *Tityus serrulatus* venom (TsV) lipids. **(A,B)** IL-1β was determined in supernatants of C57BL/6 (WT), *Cd14*^−/−^, or *Cd36^obl/obl^* cells pre-treated with or without lipopolysaccharide (LPS) (1,000 ng/mL for 4 h), followed by incubation with RPMI-I (vehicle) or TsV (50 µg/mL for 20 h). **(C,D)** C57BL/6 (WT), *Cd14*^−/−^, or *Cd36^obl/obl^* cells were incubated with crude TsV, the fatty acid-enriched fraction, or Ts1 toxin (50 µg/mL for 24 h) for IL-1β measurements. Cells from each mouse strain incubated with vehicle (medium) were used as controls [continuous lines (C57BL/6) or dashed lines (*Cd14*^−/−^ or *Cd36^obl/obl^*)]. Data are representative of two experiments (*n* = 4 in each experiment). Differences with *P* values of less than 0.05 were considered significant according to one-way analysis of variance with Bonferroni’s *post hoc* test, and error bars denote SDs. *Vehicle versus stimuli; ^$^C57BL6 versus *Cd36^obl/obl^* or *Cd14*^−/−^; ^#^TsV versus other stimuli.

In response to TsV, C57BL/6 (wild type) mouse macrophages increase IL-1β secretion *via* PGE_2_–cAMP–PKA–NFκB pathway and activation of NLRP3 inflammasome, a molecular mechanism that is repressed by LTB_4_ (3). We have previously demonstrated that CD14 induces inflammatory cytokines and PGE_2_ production in response to TsV (11), whereas CD36 has been implicated on inflammasome activation and IL-1β maturation (16). We thus postulated that CD14 and CD36 could be involved in TsV sensing and positive regulation of IL-1β production. In line with these observations, TsV-stimulated peritoneal macrophages from C57BL/6 mice upregulated *Cd14* and *Cd36* gene expression (see Figure S1A in Supplementary Material) (11). To determine whether CD14 and CD36 directly affect TsV-induced IL-1β release, we quantified IL-1β secreted by peritoneal macrophages, from C57BL/6, *Cd14*^−/−^, and *Cd36^obl/obl^* mice, stimulated with TsV or LPS; or treated with LPS and further incubated with TsV. Compared to wild-type cells, those from *Cd14*^−/−^ mice failed to produce significant amounts of IL-1β, whose levels were comparable to those of cells stimulated with LPS or with both stimuli (Figure 1A). Surprisingly, *Cd36^obl/obl^* macrophages stimulated with TsV or LPS released higher levels of IL-1β compared to wild-type cells (Figure 1B). Moreover, *Cd36^obl/obl^* cells incubated with both stimuli released similar amounts of IL-1β induced by TsV or LPS alone, suggesting that levels of secreted IL-1β reached a maximum under all conditions (ceiling effect). To confirm that the opposing roles of CD14 and CD36 were confined to the release of IL-1β, we quantified TNF-α and IL-6 secreted by TsV-stimulated macrophages. We found that both *Cd14*^−/−^ and *Cd36^obl/obl^* cells secreted less TNF-α and IL-6 than wild-type cells (see Figure S1B in Supplementary Material). These data show that both CD14 and CD36 induce pro-inflammatory cytokine response upon TsV stimulation. However, CD36 was also required to specifically repress IL-1β secretion by mouse macrophages.

CD36 functions as a scavenger receptor known to bind to fatty acids, while CD14 has also been shown to interact with this subclass of lipids (30, 41). TsV is composed by a plethora of molecules, that can be separated into a fatty acid-enriched fraction (rich in lipids and carbohydrates), or purified toxins, whereby Ts1 is the major TsV effector protein (42, 43). To understand the chemical nature of TsV effectors activating both receptors, we stimulated C57BL/6-, *Cd14*^−/−^-, and *Cd36^obl/obl^*-peritoneal macrophages with crude TsV, the fatty acid-enriched fraction, or purified Ts1. Compared to vehicle-stimulated wild-type cells, all stimuli induced IL-1β production, although crude TsV was more potent than the two isolated fractions (Figures 1C,D). Of note, neither TsV nor any of the isolated fractions induced higher IL-1β release by CD14-deficient cells (Figure 1C). TsV and Ts1 induced significant IL-1β release by *Cd36^obl/obl^* macrophages, whereas the fatty acid-enriched fraction induced similar levels to that of wild-type cells (Figure 1D). Previous work has shown that both receptors can be activated by proteins or lipoproteins (44, 45), whereas these findings indicate that Ts1 is the main TsV effector toxin inducing IL-1β secretion by mouse macrophages.

### IL-1β, cAMP, LTB_4_, and PGE_2_ Are Differentially Regulated by CD14 and CD36 in TsV-Stimulated Mouse Macrophages

**Figure 2:**
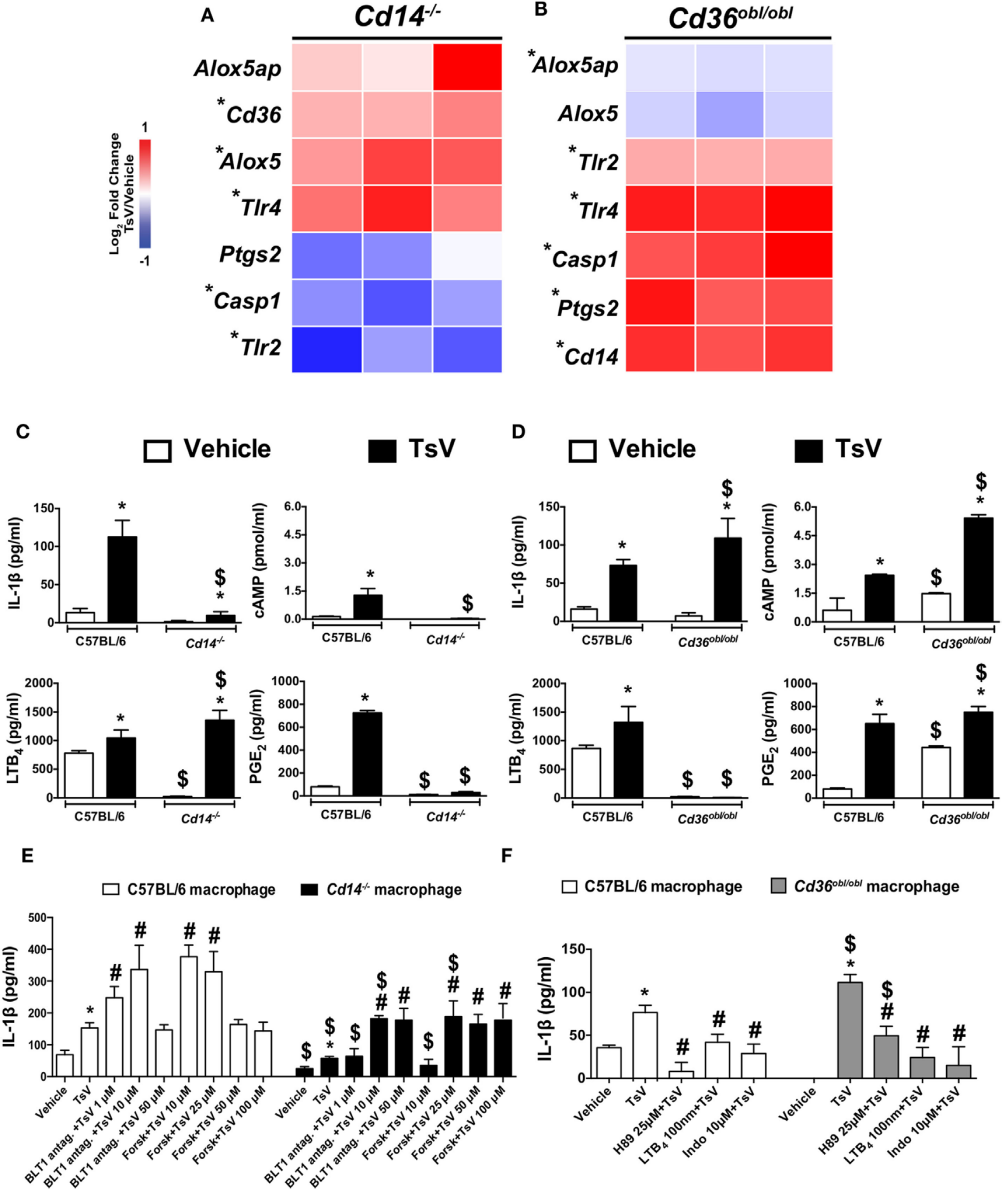
CD14 and CD36 promote differential eicosanoid metabolism to control interleukin (IL)-1β release by macrophages. **(A,B)** Peritoneal macrophages from *Cd14*^−/−^ or *Cd36^obl/obl^* mice were stimulated or not with *Tityus serrulatus* venom (TsV) (50 µg/mL) for 24 h and used to determine gene expression profiles by quantitative real-time reverse transcription polymerase chain reaction. The results were normalized to the expression of the endogenous internal control *Gapdh*. The ΔΔCt method was used for the analysis, and the data were expressed as log2 fold-change in relation to the control (vehicle) from two independent experiments (*n* = 3 in each experiment). *Vehicle versus TsV, by two-tailed Student’s *t*-test, with *P* < 0.05. **(C,D)** Peritoneal macrophages of C57BL/6, *Cd14*^−/−^, or *Cd36^obl/obl^* mice were stimulated with TsV (50 µg/mL) for 24 h, and supernatants were collected for IL-1β, leukotriene (LT)B_4_, prostaglandin (PG)E_2_, tumor necrosis factor (TNF)-α, and IL-6 quantification by enzyme-linked immunosorbent assay. In a separate set of experiments, cyclic adenosine monophosphate production was quantified at 5 min after TsV (50 µg/mL) stimulation. In all experiments, cells incubated with medium (vehicle) were used as controls. Data are representative of three independent experiments (*n* = 4 in each experiment). Significant differences are marked with symbols, and error bars denote SDs. *Vehicle versus TsV; ^#^C57BL/6 (WT) versus *Cd36^obl/obl^* or *Cd14*^−/−^. *P* < 0.05 according to one-way analysis of variance (ANOVA) with Bonferroni’s *post hoc* test. **(E)** Peritoneal macrophages from C57BL/6 or *Cd14*^−/−^ mice were pre-treated as indicated with U75302 (BLT1 antagonist, 30 min) or forskolin (Forsk, 10 min) before addition of TsV (50 µg/mL) for 24 h. **(F)** Peritoneal macrophages from C57BL/6 or *Cd36^obl/obl^* mice were pre-treated with or without H89 (25 µM, 2 h), soluble LTB_4_ (100 nM, 10 min), or indomethacin (Indo, 10 µM, 30 min). Supernatants were collected for IL-1β quantification. In all experiments, cells in medium containing the vehicle were used as controls (Vehicle). Data are representative of two independent experiments (*n* = 4 in each experiment). Significant differences (*P* < 0.05) are marked with symbols, and error bars denote SDs. *Vehicle versus TsV; ^#^TsV versus treatment; ^$^C57BL/6 versus *Cd36^obl/obl^* or *Cd14*^−/−^, according to one-way ANOVA with Bonferroni’s *post hoc* test.

Given the importance of PRRs and eicosanoid metabolism on the induction of pro-inflammatory cytokines in response to TsV, we stimulated peritoneal macrophages from *Cd14*^−/−^ and *Cd36^obl/obl^* mice with TsV, and evaluated the relative gene expression of *Cd14, Cd36, Tlr2, Tlr4, Pges2, Aloxap, Alox-5*, and *Casp1*. Of interest, *Cd36, Tlr4*, and *Alox5* expression was significantly upregulated in TsV-stimulated *Cd14*^−/−^ peritoneal macrophages, whereas *Tlr2* and *Casp1* were significantly downregulated compared to vehicle-treated cells (Figure 2A). By contrast, TsV-stimulated macrophages from *Cd36^obl/obl^* mouse exhibited significant increase on the expression of *Cd14, Tlr2, Tlr4, Pges2*, and *Casp1*, while *Alox5* and *Alox5ap* were downregulated compared to vehicle-treated cells (Figure 2B). These data suggest that after TsV engagement, CD14 and CD36 regulate each other at the transcriptional level. Importantly, the results indicate that CD14 and CD36 may affect IL-1β release by inducing PGE_2_ or LTB_4_ production, respectively.

We tested this hypothesis by stimulating *Cd14*^−/−^, *Cd36^obl/obl^*, and wild-type peritoneal macrophages with TsV to evaluate the release of IL-1β, LTB_4_, PGE_2_, and cAMP synthesis. Compared to wild-type cells, TsV-stimulated peritoneal macrophages from *Cd14*^−/−^ mouse produced 92% less IL-1β, 97% less cAMP and 96% less PGE_2_, but 30% more LTB_4_ (Figure 2C). Of note, macrophages from *Cd36^obl/obl^* mouse incubated with vehicle produced significantly higher amounts of cAMP and PGE_2_ (Figure 2D). Moreover, TsV-stimulated peritoneal macrophages from *Cd36^obl/obl^* increased IL-1β production by 49%, cAMP by 123%, and PGE_2_ by 15%, while LTB_4_ production decreased by 99% (Figure 2D). We validated these findings using pharmacological assays. First, peritoneal macrophages from *Cd14*^−/−^ mice were stimulated with TsV after pre-treatment with increasing concentrations of a BLT1 antagonist (to block signaling by LTB_4_), or forskolin (an adenylate cyclase activator, which increases cAMP levels) (3). As expected, low concentrations of BLT1 antagonist (1 µM) or forskolin (10 µM) already increased IL-1β production by wild-type cells; however, only treatment with higher concentrations of BLT1 antagonist (10 µM) and forskolin (25 µM) potentiated IL-1β production by macrophages from *Cd14*^−/−^ mouse (Figure 2E). In addition, peritoneal macrophages from *Cd36^obl/obl^* mouse were stimulated with TsV after pre-treatment with the compound H89, a PKA inhibitor, LTB_4_, and indomethacin (COX1/2 inhibitor). IL-1β production by peritoneal macrophages from C56BL/6 and *Cd36^obl/obl^* was inhibited by all compounds, but with distinct levels of magnitude (Figure 2F). Taken together, these data indicate that CD14 promotes PGE_2_, cAMP, and IL-1β release by peritoneal macrophages upon TsV challenge. By contrast, CD36 is critical for 5-LO pathway activation and downregulation of IL-1β release.

Both CD14 and CD36 play major roles as TLR co-receptors, and recruit TLR-dependent and independent signaling pathways (17). Moreover, the absence of CD14 or CD36 promotes differential gene expression of *Tlr2* and *Tlr4* in TsV-stimulated peritoneal macrophages (Figures 2A,B). To understand whether TLRs are required for responses conferred by TsV-activation of CD14 and CD36, we stimulated peritoneal macrophages from *Tlr2*^−/−^ or *Tlr4*^−/−^ mice with TsV. We measured IL-1β, PGE_2_, TNF-α, and IL-6 production, and *Ptgs2* gene expression. Compared to wild-type cells, TsV-stimulated *Tlr2*^−/−^ cells showed higher PGE_2_ production (see Figure S2A in Supplementary Material) (11) and *Ptgs2* expression (see Figure S2B in Supplementary Material). However, they released less IL-1β, TNF-α, and IL-6 (see Figures S2C–E in Supplementary Material) (11). This indicates that, in response to TsV, TLR2 partially control the IL-1β production and release by distinct mechanisms. By contrast, TsV-stimulated *Tlr4*^−/−^ macrophages released significantly less PGE_2_, IL-1β, TNF-α, and IL-6 and expressed less *Ptgs2* (see Figure S2 in Supplementary Material) (11). These results show that TLR4 is also required for PGE_2_ and IL-1β release in response to TsV, suggesting that CD14 may mediate its effects through a TLR4-dependent signaling pathway. Importantly, these results suggest that CD36 controls a regulatory mechanism that is independent of TLR2/4 signaling.

### CD14-Deficient Mice Are Resistant to TsV, While LTB_4_ Antagonist Potentiates Inflammation and Mortality

**Figure 3:**
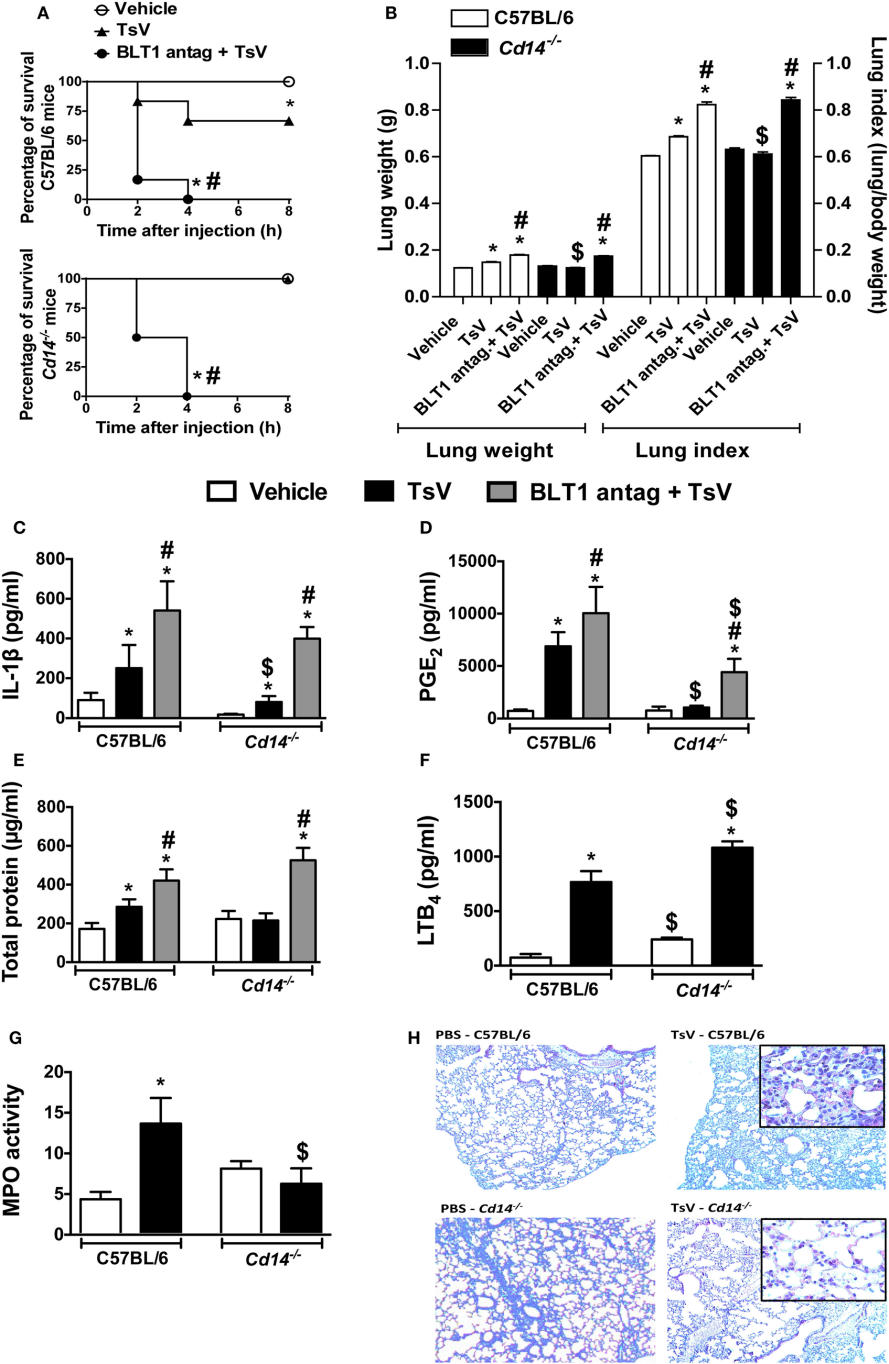
CD14 mediates *Tityus serrulatus* venom (TsV)-induced lung inflammation and mortality, which are potentiated by BLT1 antagonism. C57BL/6 (WT) mice and *Cd14*^−/−^ mice treated with the BLT1 antagonist (U-75302, 50 ng/mouse, i.n.) or vehicle were inoculated with a lethal dose of TsV (180 µg/kg, i.p.). Mice inoculated with PBS were used as controls. **(A)** Survival was monitored for 8 h. The experiment was conducted once using six mice, and error bars denote SDs. Differences with *P* values of less than 0.05 were considered significant (log-rank tests). All mice were weighed immediately before injection, and the lungs were excised instantly after animal death or from mice surviving for 8 h and weighed. **(B)** Lung indexes were calculated by determining the ratio of lung weight to body weight multiplied by 100. Lungs were then employed for analysis of **(C)** interleukin (IL)-1β, **(D)** prostaglandin (PG)E_2_, **(E)** total protein, **(F)** leukotriene (LT)B_4_ concentrations, and **(G)** myeloperoxidase (MPO) activity. The experiment was conducted once using six mice per group, and error bars denote SDs. *PBS versus vehicle plus TsV or BLT1 antagonist plus TsV; ^#^vehicle plus TsV versus BLT1 antagonist plus TsV; ^$^C57BL/6 mice versus *Cd14*^−/−^ mice. Differences with *P* values of less than 0.05 were considered significant according to one-way analysis of variance with Bonferroni’s *post hoc* test. **(H)** Lungs were prepared for staining with hematoxylin and eosin (H&E) for histological analysis (*n* = 4). Histopathology of lung tissues was examined [100× and 1,000× (inset) magnification].

Envenomation with TsV culminates in lung inflammation and edema, resultant from the inflammatory effects of IL-1β (3). In line with these observations, and based on the results obtained *in vitro*, we investigated whether CD14 contributes to TsV-induced lung inflammation and mortality *in vivo*. Wild-type and *Cd14*^−/−^ mice were weighed and inoculated intraperitoneally (i.p.) with a lethal dose of TsV (180 µg/kg), and mortality was observed for 8 h (Figure 3A). An additional group of lethally envenomed mice from both strains was treated with BLT1 antagonist U75302. After envenomation, 100% of CD14-deficient animals survived, in contrast to 67% of wild-type animals (Figure 3A). We assessed lung tissues after animal death and calculated the lung index, which reflects lung edema. Interestingly, TsV-inoculated *Cd14*^−/−^ mice exhibited reduced lung edema compared to C57BL/6 mice (Figure 3B). Moreover, lungs of TsV-inoculated *Cd14*^−/−^ mice exhibited reduced levels of IL-1β (Figure 3C) and PGE_2_ (Figure 3D); but showed similar amounts of protein in the parenchyma (Figure 3E) and released higher levels of LTB_4_ (Figure 3F). Unexpectedly, the lungs of vehicle-inoculated *Cd14*^−/−^ mice showed abundant neutrophils compared to wild-type mice, as demonstrated by MPO quantification (Figure 3G). However, the cellular content was not affected after TsV inoculation (Figure 3G). These findings were confirmed by histological analysis of the lungs (Figure 3H). As predicted, treatment of envenomed mice with the BLT1 antagonist U75302 worsened inflammation, regardless the mouse strain; 100% of mice from both strains died within 4 h (Figure 3A), released higher amounts of IL-1β and PGE_2_ in the lung (Figures 3C,D), and developed stronger edema (Figures 3B,E). These findings support our previous observations that in this context, LTB_4_ binding to BLT1 exhibits a regulatory activity (3). Overall, these results demonstrate that CD14 is an inflammatory receptor that contributes to lung inflammation and mortality induced by TsV *via* PGE_2_–cAMP–IL-1β release.

### CD36-Deficient Mice Are Susceptible to TsV, and LTB_4_ Treatment Rescues Fatal Outcomes

**Figure 4:**
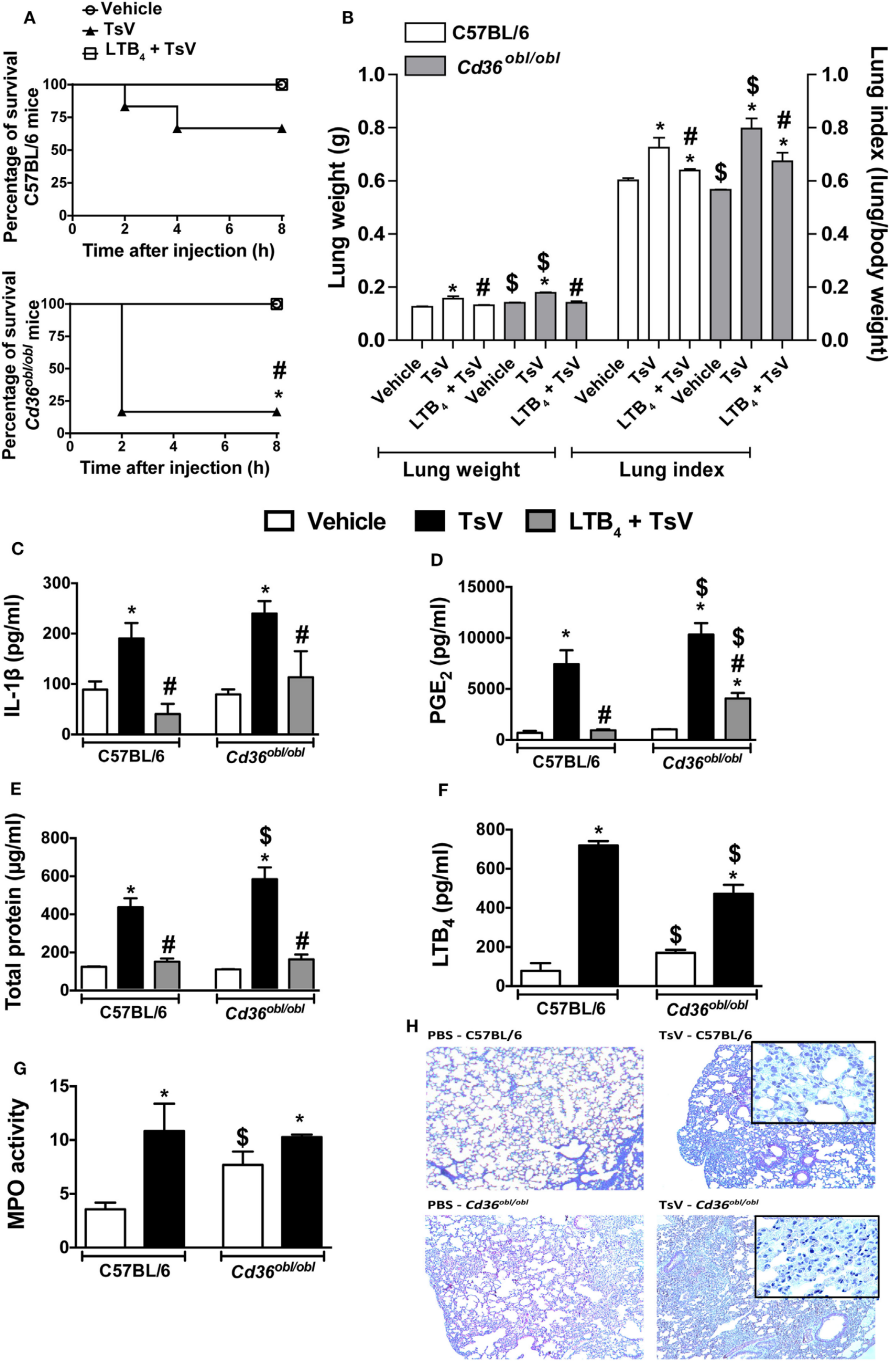
CD36 controls a regulatory mechanism that depends on leukotriene (LT)B_4_ production. C57BL/6 (WT) and *Cd36^obl/obl^* mice treated with exogenous LTB_4_ (50 ng/mouse, i.n.) or vehicle were inoculated with a lethal dose of *Tityus serrulatus* venom (TsV) (180 µg/kg, i.p.). Mice inoculated with PBS were used as controls. **(A)** Survival was monitored for 8 h. The experiment was conducted once using six mice, and the error bars denote SDs. The differences were considered significant when the *P* value was less than 0.05 according to log-rank tests. All mice were weighed immediately before the injections, and the lungs were excised instantly after animal death or from mice surviving until 8 h and weighed. **(B)** Lung indexes were calculated by determining the ratio of lung weight to body weight multiplied by 100. Lungs were then employed for analysis of **(C)** interleukin (IL)-1β, **(D)** prostaglandin (PG)E_2_, **(E)** total protein, **(F)** LTB_4_ concentrations, and **(G)** myeloperoxidase (MPO) activity. The experiment was conducted once with six mice per group, and error bars denote SDs. *PBS versus vehicle plus TsV or LTB_4_ plus TsV; ^#^vehicle plus TsV versus LTB_4_ plus TsV; ^$^C57BL/6 mice versus *Cd36^obl/obl^* mice. These differences were considered significant when the *P* value was less than 0.05 according to one-way analysis of variance with Bonferroni’s *post hoc* test. **(H)** Lungs were prepared for staining with hematoxylin and eosin (H&E) for histological analysis (*n* = 4). The histopathology of lung tissues was examined [100× and 1,000× (inset) magnification].

In response to TsV, CD36-deficient mouse macrophages significantly increase IL-1β secretion, suggesting that upon scorpion envenomation, these animals might develop exacerbated inflammatory responses. Indeed, *Cd36^obl/obl^* mice were highly susceptible to TsV compared to wild-type mice; 83% of deficient mice died in the first 2 h, in contrast to only 67% of C57BL/6 mice within 8 h (Figure 4A). Interestingly, exogenous administration of LTB_4_ abrogated TsV-induced mortality in both mice strains (Figure 4A). *Cd36^obl/obl^* mice exhibited robust lung edema, measured by lung weight and index (Figure 4B). Consistent with the augmented sensitivity of CD36-deficient mice to TsV, the concentrations of IL-1β (Figure 4C), PGE_2_ (Figure 4D), and total protein (Figure 4E) were significantly higher, whereas that of LTB_4_ (Figure 4F) was significantly lower compared to C57BL/6 animals. Interestingly, *Cd36^obl/obl^* vehicle-inoculated animals showed increased numbers of neutrophils (Figures 4G,H). TsV inoculation induced recruitment of these granulocytes into the lung, whereas a robust increase in MPO was observed in C57BL/6 animals (Figure 4G). These results were confirmed by lung histology (Figure 4H), showing that CD36 deficiency resulted in a pro-inflammatory phenotype and that leukocytes accumulated in the parenchyma. As predicted, administration of exogenous LTB_4_ inhibited IL-1β, PGE_2_, protein extravasation, and edema in both strains of mice (Figures 4B–E), corroborating the anti-inflammatory activity of this 5-LO catabolite. These data confirm the regulatory role of CD36 receptor, protecting the host from excessive inflammation and mortality.

### IL-1β Release by TsV-Stimulated Human PBMCs Is Controlled by LTB_4_ and PGE_2_ Balance

**Figure 5:**
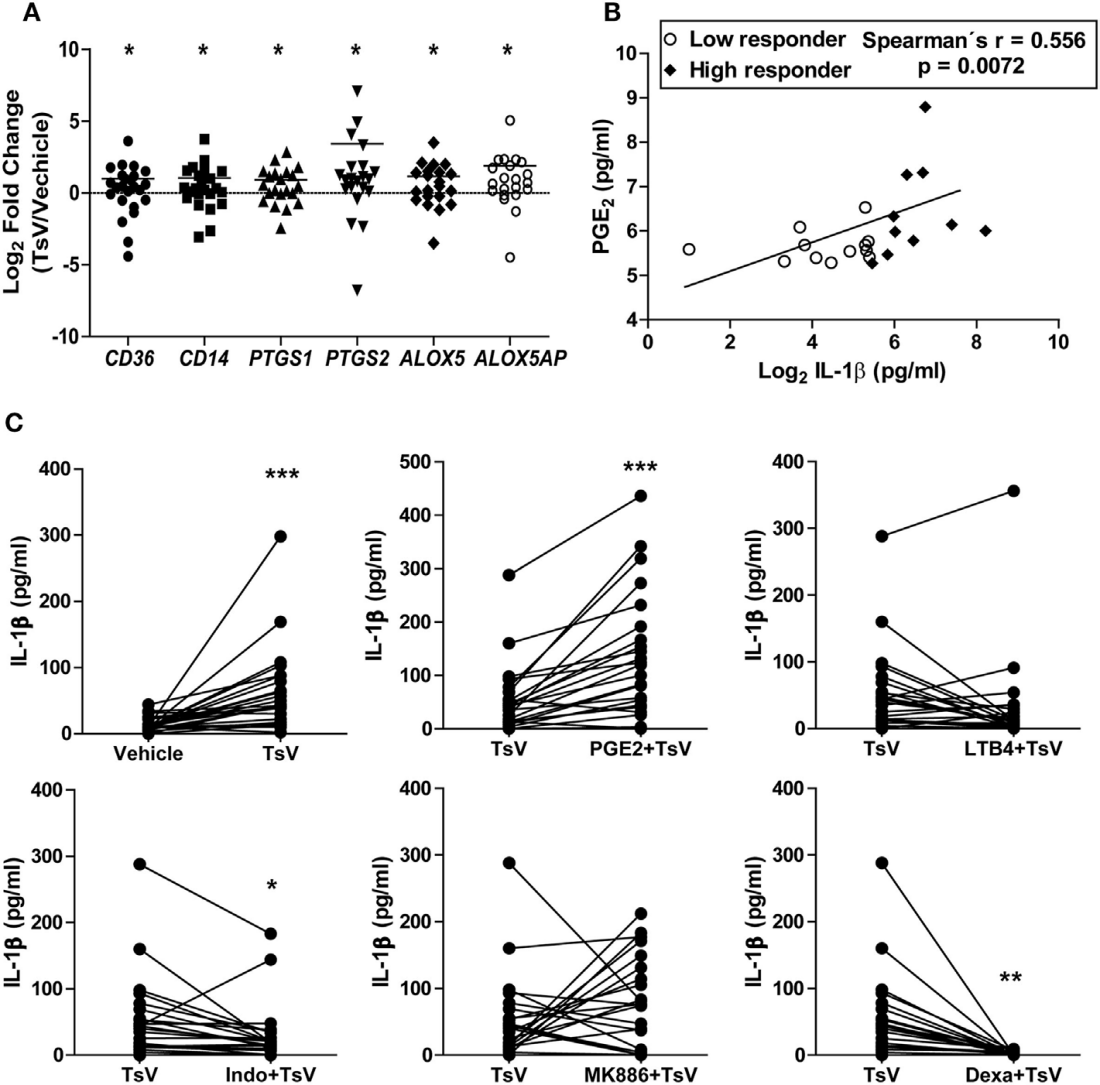
Prostaglandin (PG)E_2_ boosts *Tityus serrulatus* venom (TsV) induced interleukin (IL)-1β release by human peripheral blood mononuclear cells (PBMCs). **(A)** PBMCs from healthy individuals were stimulated with or without TsV (50 µg/mL) for 24 h, and the cell lysates were collected for gene expression analyses of *CD36, CD14, PTGS1, PTGS2*, ALOX5, and *ALOX5AP* by quantitative real-time reverse transcription polymerase chain reaction (*n* = 24). **(B)** Culture supernatants were used for IL-1β quantification by enzyme-linked immunosorbent assay or PGE_2_ by liquid chromatography-tandem mass spectrometry (LC-MS/MS). Correlation analysis was performed with Spearman’s correlation method (*n* = 24). **(C)** PBMCs from healthy individuals were pre-treated with or without PGE_2_ (10 µM, 10 min, *n* = 24), leukotriene (LT)B_4_ (100 nM, 10 min, *n* = 24), indomethacin (Indo, 10 µM, 30 min, *n* = 24), MK886 (10 µM, 30 min, *n* = 22), or dexamethasone (Dexa, 0.1 µM, 1 h, *n* = 12) before addition of TsV (50 µg/mL) for 24 h. In all experiments, unstimulated (vehicle) cells were used as controls. Paired two-tailed Student’s *t*-test was used to evaluate differences. Significance is given as *P < 0.05, **P < 0.01, and ***P < 0.001.

Together with our previous observations (3), data presented here provide compelling evidences for molecular mechanisms controlling TsV-induced inflammatory responses and mortality in mice. However, the mechanisms that drive inflammatory cascades and IL-1β release by human cells during scorpion envenomation have not been investigated. Notably, envenomed patients exhibit high levels of systemic sCD14 compared to healthy controls (see Figure S3A in Supplementary Material), indicating that monocytes are robustly activated after scorpion sting (27). TsV stimulation of whole blood, from a subset of 10 healthy subjects, revealed decreased cell membrane expression of CD14 measured by flow cytometry (see Figure S3B in Supplementary Material), while CD36 expression remained unaltered (see Figure S3C in Supplementary Material). We obtained further insights by isolating PBMCs from 24 healthy subjects, which were later incubated with TsV. PBMCs stimulated with TsV increased gene expression of *CD36, CD14, PTGS1, PTGS2, ALOX5*, and *ALOXAP* compared to vehicle-stimulated cells (Figure 5A). Moreover, we identified a significant positive correlation between levels of PGE_2_ and IL-1β secreted by TsV-stimulated human PBMCs (Figure 5B). We then proceeded by pre-treating the cells with vehicle, PGE_2_, LTB_4_, the COX1/2 inhibitor indomethacin, or the 5-lipoxygenase-activating protein (FLAP) inhibitor MK886; followed by stimulation with TsV for 24 h and IL-1β quantification. Compared to vehicle-treated PBMCs, TsV induced significant IL-1β release (range: 13–298 pg/mL) (Figure 5C). Compared to TsV-stimulated PBMCs, pre-treatment with PGE_2_ increased IL-1β release significantly, while indomethacin and dexamethasone significantly decreased IL-1β release (Figure 5C). After TsV stimulation, PBMCs also released increased amounts of IL-6, whereas only 50% of volunteers showed increased TNF-α when compared with cells incubated with vehicle (see Figures S3D,E in Supplementary Material).

**Figure 6:**
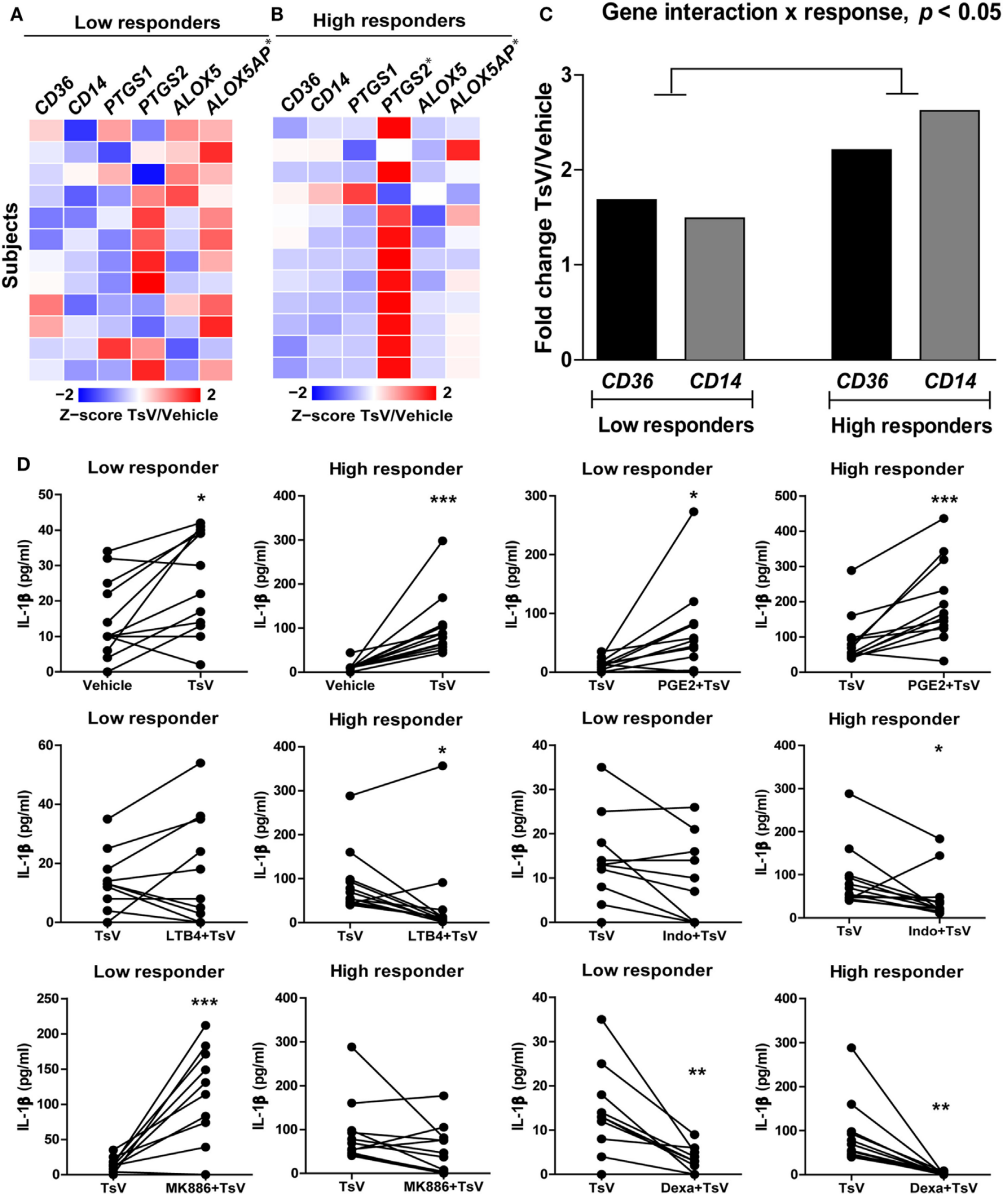
Differential eicosanoid metabolism determines the levels of interleukin (IL)-1β released by *Tityus serrulatus* venom (TsV)-stimulated human PBMCs. Individuals were classified into low (*n* = 12) or high (*n* = 12) responders based on the median value IL-1β upon stimulation with or without TsV (50 µg/mL) for 24 h. **(A,B)** Cell lysates were collected for gene expression analyses of *CD36, CD14, PTGS1, PTGS2*, ALOX5, and *ALOX5AP* by quantitative real-time reverse transcription polymerase chain reaction. **(C)** Two-way analysis of variance of gene expression of *CD36* and *CD14* between low and high responders. **(D)** PBMCs from low or high responders were pre-treated with or without prostaglandin (PG)E_2_ (10 µM, 10 min, *n* = 24), leukotriene (LT)B_4_ (100 nM, 10 min, *n* = 24), indomethacin (Indo, 10 µM, 30 min, *n* = 24), MK886 (10 µM, 30 min, *n* = 22), or dexamethasone (Dexa, 0.1 µM, 1 h, *n* = 12) before addition of TsV (50 µg/mL) for 24 h. In all experiments, unstimulated (vehicle) cells were used as controls. Paired two-tailed Student’s *t*-test was used to evaluate differences. Significance is given as *P < 0.05, **P < 0.01, and ***P < 0.001.

We observed highly variable levels of IL-1β released by human PBMCs. We thus hypothesized that a differential activity of enzymes involved in the metabolism of LTB_4_ and PGE_2_ could explain these variations. Therefore, using the median IL-1β concentration value (42 pg/mL of IL-1β per 5 × 10^5^ PBMCs), we classified volunteers into low or high responders based on their ability to release IL-1β upon TsV stimulation. As expected, PBMCs from high responders secreted increased amounts of IL-1β and PGE_2_ (see Figures S3F,G in Supplementary Material). Furthermore, the significance in expression of *CD36, CD14, PTGS1, PTGS2, ALOX5*, and *ALOXAP* changed according to the group of subjects relative to vehicle-stimulated PBMCs. Low responders only exhibited significant increase in expression of *ALOXAP* (Figure 6A), while high responders exhibited significant increase in expression of both *PTGS2* and *ALOXAP* (Figure 6B). We were unable to identify significant differences in gene expression between low and high responders by comparing difference between expression of single genes. However, we identified significant difference between low and high responders on the interaction term of CD14*CD36 (*F*_1.22_ = 4.61, *P* = 0.043) (Figure 6C). This suggests that after stimulation with TsV, there is a significant association between gene expression of CD14 and CD36, which correlates with the magnitude of IL1-β release. Irrespective of classification group, gene expression was not significantly correlated with IL1-β quantification, possibly due differential dynamics on transcription and translation after TsV stimulation. However, correlation networks based on the expression of the studied genes demonstrate different association patterns between low and high responders (see Figure S3H in Supplementary Material). For example, high responders exhibited weaker correlations of *CD36* with *ALOXAP* and *PTGS2* compared to low responders (see Figure S3H in Supplementary Material).

Both groups (high and low responders) exhibited significant increase in IL-1β release compared to vehicle-stimulated PBMCs (Figure 6D). Compared to TsV-stimulated PBMCs, pre-treatment with PGE_2_ induced significant increase in IL-1β release, while dexamethasone decreased IL-1β release in both groups (Figure 6D). Pre-treatment with LTB_4_ or indomethacin significantly reduced IL-1β release by high responders (Figure 6D). However, treatment with the MK886 significantly increased IL-1β release only in low responders (Figure 6D). These results support a mechanism in which PGE_2_ boosts IL-1β production and release in response to TsV, while levels of IL-1β are determined by differential activity of eicosanoid metabolism in low and high responders.

## Discussion

In this study, we demonstrated the pivotal, but contrasting roles of CD14 and CD36 receptors in the regulation of LTB_4_, PGE_2_, and cAMP production, and control of IL-1β-mediated inflammation. Moreover, we confirmed that human cell responses to TsV are driven by mechanisms compatible with those observed in mice. These findings are particularly relevant in translational medicine. Scorpion envenomed patients exhibit clinical manifestations varying from local lesions to severe lung inflammation and cardiogenic shock, which can be fatal (20–22, 43, 46). TsV induces substantial release of inflammatory cytokines. However, the balance between 5-LO and COX1/2 catabolites control the levels of inflammasome-derived IL-1β, a potential determinant of human envenomation severity, as observed in mice (3). We confirmed that human PBMCs responded to TsV by releasing IL-1β, although levels of IL-1β production varied greatly. In almost all PBMCs, PGE_2_ increased IL-1β release, with substantial variations in the LTB_4_ response. However, when donors were classified into low and high cytokine responders, we found that high IL-1β responders were more sensitive to indomethacin and LTB_4_ than low responders, and low IL-1β responders were more responsive to MK886 than high responders. Thus, although PGE_2_ boosts IL-1β production in human cells, levels of this cytokine seem to be determined by the differential activity of enzymes involved in eicosanoid metabolism. To the best of our knowledge, these findings are the first to provide mechanistic insights into human responses to scorpion envenomation.

CD14 activation by LPS induces microsomal PGE synthase-1, PGE_2_ production, and skin edema formation *via* a mechanism that is independent of TLR signaling, but dependent on the transcriptional activator nuclear factor of activated T cells (24). However, our data suggest that CD14 induces PGE_2_ and IL-1β release by a pathway that is at least partially dependent on TLR4 signaling. PGE_2_ signals through interaction with four G protein-coupled receptors, EP1-EP4. While EP2 and EP4 activation by PGE_2_ promotes suppressive effects *via* cAMP/PKA/CREB pathway (47), TsV induces a different molecular signaling pathway that culminates with pro-inflammatory cytokine production *via* cAMP/PKA/NF-κB (3, 11). Interestingly, PGE_2_ induces CD14 expression in macrophages *via* cAMP/PKA (48), suggesting a regulatory loop between cAMP and CD14, and control over IL-1β-mediated inflammation. *Cd14*^−/−^ derived peritoneal macrophages produce more LTB_4_ than wild-type cells when stimulated with TsV (11), while the effects of U75302, a BLT1 antagonist, or forskolin (a PKA activator), were only observed using higher concentrations of these compounds. This suggests that *Cd14*^−/−^ cells expressed less BLT1 and/or more BLT2 receptors. The expression of BLT1 is higher in CD14^+^ monocytes (49). In the lungs, TsV-inoculated CD14-deficient mice also released significantly more LTB_4_ than C57BL/6 mice, and the concentrations of IL-1β and PGE_2_ were close to zero. Moreover, higher amounts of LTB_4_ in *Cd14*^−/−^ animals protected the mice from TsV-induced lung edema and death. Treatment of *Cd14*^−/−^ with the BLT1 antagonist blocked these effects, confirming that the anti-inflammatory effects of this eicosanoid were mediated by BLT1 receptor. Notably, despite the higher concentration of LTB_4_ in the lungs of *Cd14*^−/−^ animals, TsV inoculation did not enhance the recruitment of neutrophils to the lungs and MPO activity was similar to vehicle-inoculated *Cd14*^−/−^ animals. LTB_4_ is widely recognized as a neutrophil chemoattractant and activator (50, 51), but our results suggest that during TsV-induced inflammation, this lipid mediator does not contribute to this phenomenon, instead neutrophils are recruited *via* IL-1β (52). Whether neutrophils from *Cd14*^−/−^ mice express less BLT1 and/or more BLT2 is also an important point. However, our results suggest that CD14 signaling may differentially regulate BLT1 expression depending on the leukocyte type. Supporting this hypothesis, LPS priming of neutrophils and later stimulation with FMLP, C5a, or IL-8 increases their production of 5-LO metabolites by a mechanism that depends on CD14 (53). However, further investigation is needed to determine if and how CD14 regulates differential BLT1/2 expression. Although the critical roles of CD14 (54, 55) and IL-1β secretion (3, 56) have been investigated during lung inflammation, the results presented here demonstrate unrecognized relationships between CD14, IL-1β, cAMP, and bioactive lipids.

Recently, CD36 has been described a central coordinator of NLRP3 inflammasome activation and IL-1β release during sterile inflammation (16). In addition, CD36 mediates phagocytosis of apoptotic cells (57, 58) that results in PGE_2_ release (59). We thus hypothesized that CD36 would contribute to the activation of NLRP3 and increase IL-1β secretion during scorpion envenomation. Nevertheless, we found a paradoxical role of this receptor. While CD36 mediates inflammatory signaling that results in the induction of TNF-α and IL-6, it also specifically regulates the release of IL-1β. Indeed, *Cd36* deficiency abrogated LTB_4_ production, resulting in increased production of PGE_2_, cAMP, and IL-1β by TsV-stimulated macrophages. Furthermore, *in vivo* data confirmed that the inflammatory phenotype of *Cd36^obl/obl^* mice was due impaired activation of the 5-LO pathway and decreased production of LTB_4_. Inoculation of a lethal dose of TsV in *Cd36^obl/obl^* mice resulted in robust release of PGE_2_ and IL-1β, with intense lung edema and cell infiltration. Intranasal LTB_4_ administration suppressed PGE_2_ and IL-1β release, blocked lung edema, and prevented death in *Cd36^obl/obl^* mice, further validating an anti-inflammatory function of this eicosanoid. Importantly, expression of the CD36 receptor is induced during higher activity of 5-LO pathway (60), corroborating a direct relationship between CD36 and LTB_4_ metabolism. CD36 transfected cells exposed to different bacteria induce JNK signaling that culminates in IL-8 and IL-6 production, while NF-κB inhibition does not seem to influence this process (61). Interestingly, monocyte-derived macrophages from CD36-deficient humans secrete less TNF-α and IL-1β when stimulated with oxidized low-density lipoproteins or thrombospondin-1 (62). Previous studies have also shown that CD36-deficient bone marrow-derived macrophages or peritoneal macrophages produce less IL-1β when incubated with particulate ligands (16). Multiple factors may contribute to the opposing responses mediated by CD36, such as the nature of the ligands (soluble or particulate), the activation/participation other PRRs, the presence/absence of cofactors in the microenvironment, such as ATP and LPS (16); or even the enhanced CD36 expression mediated by activation of 5-LO pathway (60). Although CD36 induces LTB_4_ production by mouse macrophages *in vitro*, our findings show LTB_4_ is also released through CD36-independent mechanism(s) that is, however, unable to contain the TsV-induced inflammation. Neutrophil is an important LTB_4_ source (63). MPO quantification and lung tissue histology demonstrated that *Cd36^obl/obl^* mice exhibited a robust neutrophil infiltration in the lungs, which can be responsible for LTB_4_ production after envenomation.

**Figure 7:**
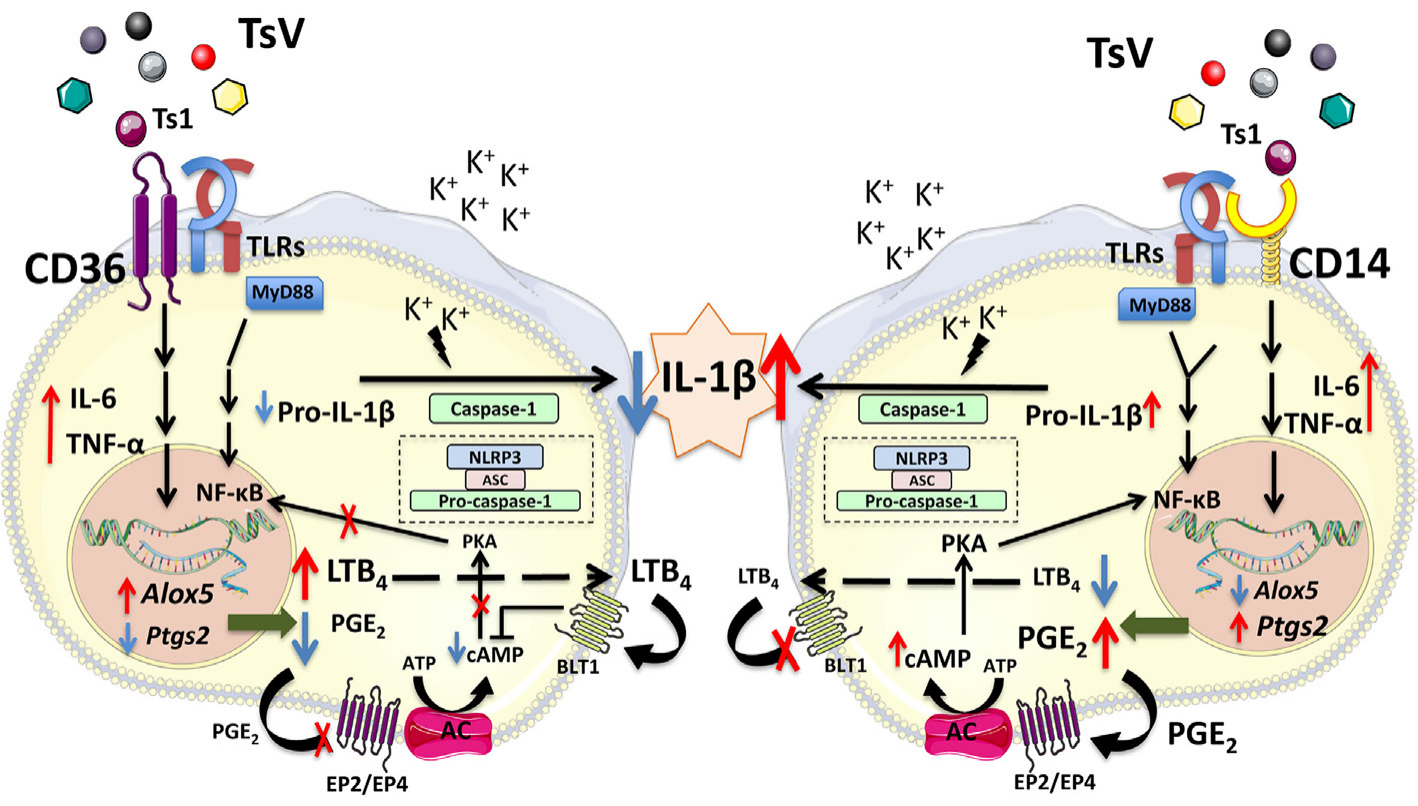
CD14 and CD36 receptors control differential eicosanoid metabolism and inflammation. *Tityus serrulatus* venom activates the NLRP3 inflammasome in macrophages, which requires potassium (K^+^) efflux, and results in interleukin (IL)-1β release by mouse peritoneal macrophages. This phenomenon is dependent on NF-kB activation, which is amplified by PGE_2_ and repressed by leukotriene (LT)B_4_ (3). Here, we show that upon TsV recognition, the receptor CD14 upregulates the *Ptgs2*/PGE_2_/cyclic adenosine monophosphate (cAMP) axis and amplification of IL-1β production and release. By contrast, CD36 shunts eicosanoid metabolism toward LTB_4_ production. This process results in decreased cAMP levels, repressing protein kinase A and NF-kB activation, which reduces IL-1β secretion and inflammation. We propose that the balance in eicosanoid metabolism, controlled by CD14 and CD36, regulates the amount of released IL-1β and dictates the outcome after scorpion envenomation.

In summary, our results demonstrate that CD14 and CD36 are important PRRs that sense scorpion venom, but exhibit opposing functions on the control of IL-1β release and inflammation (Figure 7). Upon TsV engagement, CD14 promotes the inflammatory response *via* PGE_2_/cAMP/IL-1β production, leukocyte recruitment, and edema formation, while downregulating the 5-LO pathway activity and LTB_4_ release. By contrast, CD36 plays a dual role by promoting inflammatory cytokines such as TNF-α and IL-6, while repressing IL-1β release. Specifically, it mediates LTB_4_ production, which in turn decreases cAMP production to downregulate COX2/PGE_2_. In consequence, IL-1β levels decrease, directly impacting TsV-induced mortality due reduced lung inflammation and edema. CD14 and CD36 are potential therapeutic targets for several inflammatory diseases. Therefore, our findings have strong implications to the full understanding of the functions exerted by these receptors, and thus rational for drug design. Future investigations will be necessary to confirm that the influence of CD14 and CD36 interplay on differential eicosanoid metabolism and IL-1β release regulation is not restricted to scorpion envenomation.

## Ethics Statement

*Ethics statement involving human subjects*: This study was carried out in accordance with the recommendations of the National Committee for Research Ethics (CONEP). The protocol was approved by the School of Pharmaceutical Sciences of Ribeirão Preto Human Research Ethics Committee (protocol #54426115.1.0000.5403) and Clinics Hospital of Ribeirão Preto Medical School Research Ethics Committee (protocol #54426115.1.3001.5440). All subjects gave written informed consent in accordance with the Declaration of Helsinki. *Ethics statement involving animal subjects*: This study was carried out in accordance with the recommendations of the National Council for Animal Experimentation Control (CONCEA). The protocol was approved by the Animal Care Committee of the Campus of Ribeirão Preto (PCARP) at the University of São Paulo, Ribeirão Preto, Brazil (protocol #14.1.272.53.7).

## Author Contributions

KZ designed and performed the experiments, analyzed data, and wrote the manuscript. LG analyzed data and wrote the manuscript. CS designed and performed the experiments, analyzed the data, and revised the manuscript. AFM performed experiments. PC, AKM, and VB conducted clinical evaluation of envenomed patients, collected plasma samples, and discussed hypothesis. IG and FG analyzed data and revised the manuscript. EA and KB provided the scorpion venom and discussed the hypotheses. LF conceived and supervised the project, designed the experiments, interpreted the data, and wrote the manuscript. All authors read and approved the final manuscript.

## Conflict of Interest Statement

The authors declare that the research was conducted in the absence of any commercial or financial relationships that could be construed as a potential conflict of interest.

## Supplementary Material

Figure S1 Shows the gene expression of Cd14 and Cd36 in C57BL/6 peritoneal macrophages stimulated with *T. serrulatus* venom (TsV), and tumor necrosis factor (TNF)-α and IL-6 quantification in C57BL/6, Cd14^−/−^, and Cd36^obl/obl^ peritoneal macrophages stimulated with TsV.

Figure S2 Shows gene expression of Ptgs2, and prostaglandin (PG)E_2_, IL-1β, TNF-α, and IL-6 quantification in Tlr2^−/−^ and Tlr4^−/−^ peritoneal macrophages stimulated with TsV.

Figure S3 Shows soluble CD14 quantification in plasma of envenomed patients and controls; mean fluorescence intensity (MFI) of CD14 and CD36 cell membrane expression in healthy human whole blood stimulated or not with TsV; PGE_2_, IL-1β, TNF-α, and IL-6 quantification in healthy human peripheral blood mononuclear cells (PBMC) stimulated or not with TsV; and correlation networks between gene expression of PTGS2, CD14, CD36, toll-like receptor (TLR)2, TLR4, ALOX5AP, ALOX-5, and CASP1 and IL-1β concentration in healthy human PBMC stimulated or not with TsV.

Table S1 Shows demographic data for human subjects.

Table S2 Shows hematological parameters of healthy human subjects.
