# Supplementary material for: CD36 Shunts Eicosanoid Metabolism to Repress CD14 Licensed Interleukin-1β Release and Inflammation
Source: Front Immunol. 2018 Apr 27;9:890. doi: 10.3389/fimmu.2018.00890 (PMC5934479; doi:10.3389/fimmu.2018.00890)
Supplement: Figure S1 — Shows the gene expression of Cd14 and Cd36 in C57BL/6 peritoneal macrophages stimulated with T. serrulatus venom (TsV), and tumor necrosis factor (TNF)-α and IL-6 quantification in C57BL/6, Cd14−/−, and Cd36obl/obl peritoneal macrophages stimulated with TsV. [file Image_1.PDF]

Supplementary Material

CD36 shunts eicosanoid metabolism to repress CD14 licensed interleukin-1β release and inflammation

Karina F. Zoccal[1], Luiz G. Gardinassi[1], Carlos A. Sorgi[1], Alyne F. G. Meirelles[1], Karla C. F. Bordon[2], Isaias Glezer[3], Palmira Cupo[4], Alessandra K. Matsuno[4], Valdes R. Bollela[5], Eliane C. Arantes[2], Francisco S. Guimarães[6], & Lúcia H. Faccioli[1*]* Correspondence: Corresponding

*** Correspondence:**
Lúcia Helena Faccioli, PhD
faccioli@fcfrp.usp.br

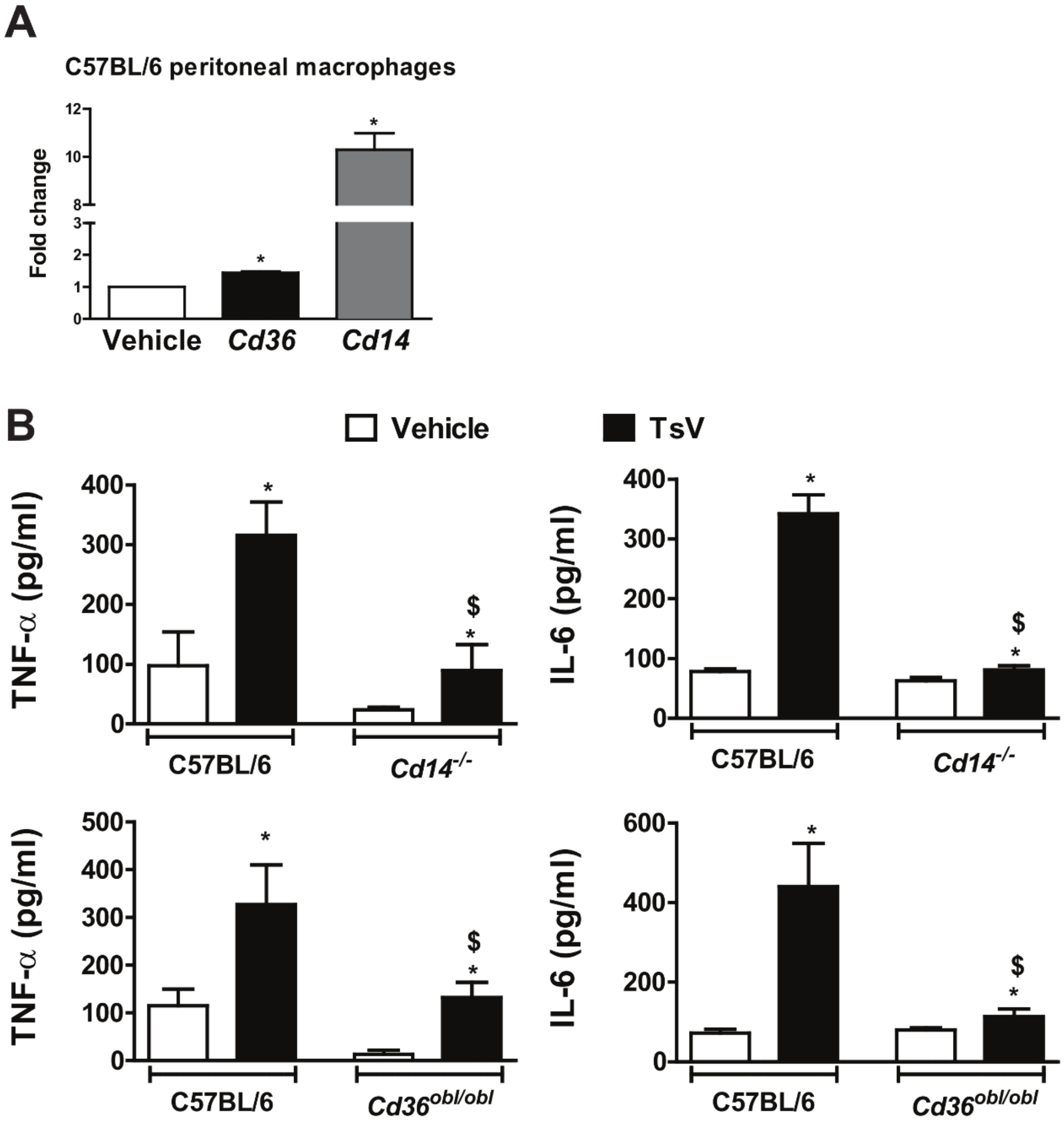

Fig S1 – Mouse macrophages express *Cd14* and *Cd36* in response to TsV, which control the production of inflammatory cytokines. (A) *Cd36* and *Cd14* mRNA expression in C57BL/6 (WT) peritoneal macrophages was determined following TsV stimulation (50 µg/mL for 4 h) by qRT-PCR. The fold change of mRNA expression was determined in comparison with cells incubated with medium alone. Data are representative of two independent experiments (*n* = 4 in each experiment), and differences were analyzed by two-tailed Student's *t*-test. **(B)** Peritoneal macrophages of C57BL/6, *Cd36*$^{obl/obl}$, or *Cd14*$^{-/-}$ mice were stimulated with TsV (50 µg/mL) for 24 h, and supernatants were collected for TNF-α, and IL-6 quantification by ELISA. In all experiments, cells incubated with medium (vehicle) were used as controls. Data are representative of three independent experiments (*n* = 4 in each experiment). Significant differences are marked with symbols, and error bars denote SDs.

*Vehicle *versus* TsV; $^{\$}$C57BL/6 (WT) *versus* *Cd36*$^{obl/obl}$ or *Cd14*$^{-/-}$. $P < 0.05$ according to one-way ANOVA with Bonferroni's post-hoc test.

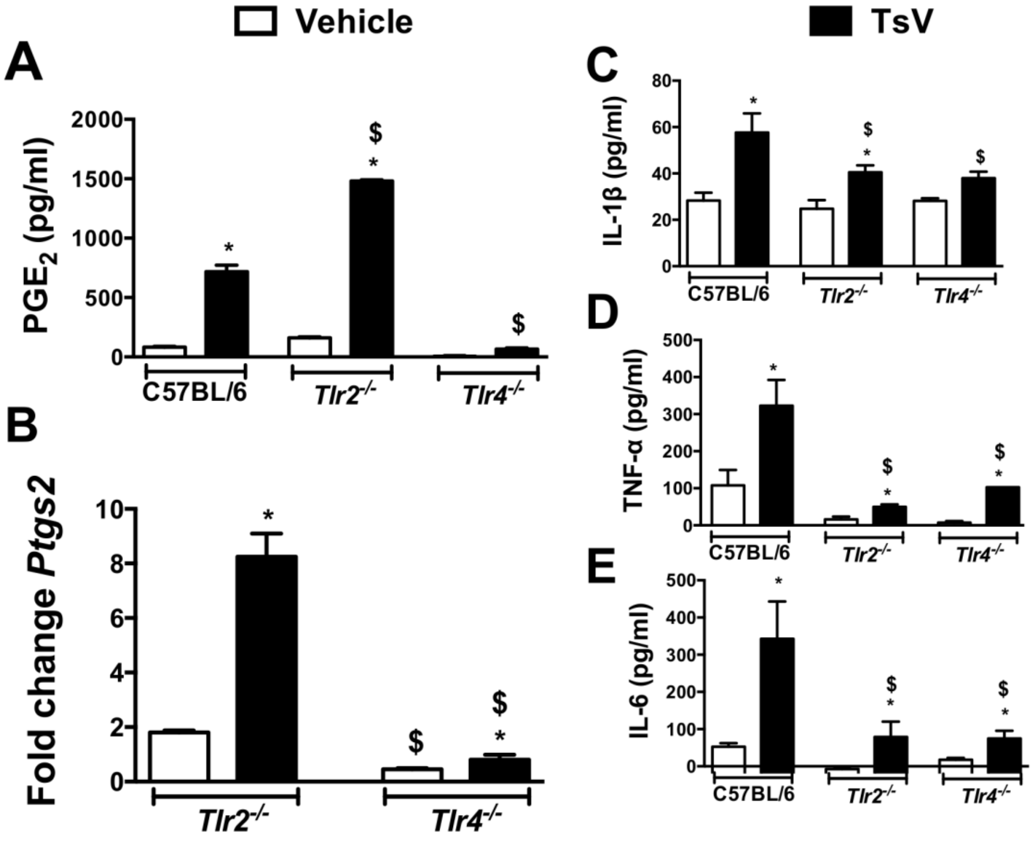

Fig. S2 – Impact of *Tlr2* or *Tlr4* deficiency on the production of inflammatory mediators. Peritoneal macrophages from C57BL/6, *Tlr2*$^{-/-}$, or *Tlr4*$^{-/-}$mice were incubated with TsV (50 µg/mL) or vehicle for 24 h. Supernatants were collected for (**A**) PGE_2, (**C**) IL-1β, (**D**) TNF-α, and (**E**) IL-6 quantification by ELISA, and (**B**) *Tlr2*$^{-/-}$ or *Tlr4*$^{-/-}$ cells were used for determination of *Ptgs2* mRNA by qRT-PCR. The fold change increase in mRNA was compared with expression in vehicle-treated cells. Data are representative of two independent experiments ($n = 4$ in each experiment). Differences with P values of less than 0.05 were considered significant according to one-way ANOVA with Bonferroni's post-hoc test, and error bars denote SDs. *Vehicle *versus* TsV; $^{\$}$C57BL/6 *versus* *Tlr2*$^{-/-}$ or *Tlr4*$^{-/-}$.

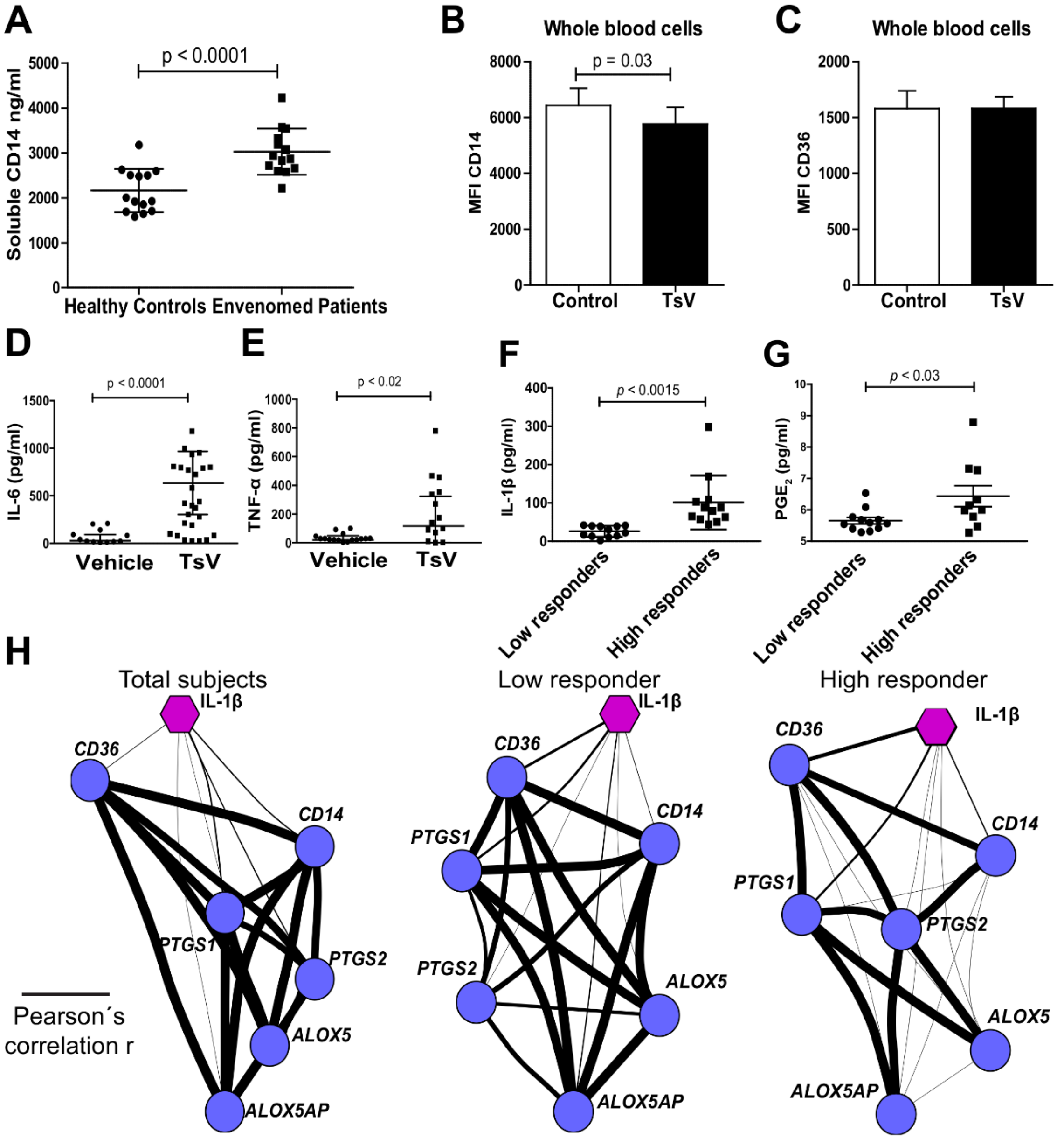

Fig. S3 – Scorpion envenomation induces inflammatory mediators in humans. (A) Serum was collected from envenomed patients and soluble CD14 was measured by ELISA. **(B-C)** Whole blood was collected from healthy individuals, stimulated with or without TsV (50 µg/mL) for 4h, and CD14 or CD36 expression was evaluated by flow cytometry. **(D-E)** PBMCs from healthy individuals were stimulated with or without TsV (50 µg/mL) for 24 h, and culture supernatants were used for TNF-α, and IL-6 quantification by ELISA. **(F-G)** Individuals were classified into low or high responders based on the median value IL-1β upon stimulation with or without TsV (50 µg/mL) for 24 h. IL-1β was quantified by ELISA and PGE_2 was quantified by liquid chromatography-mass spectrometry. Categorical comparisons were evaluated by two-tailed Student's *t*-test. **(H)** Correlation networks

including levels of IL-1β and gene expression of *CD36*, *CD14*, *PTGS1*, *PTGS2*, ALOX5 and *ALOX5AP*. Edges represent Pearson´s correlation coefficients.

Table S1. Demographic data for human blood donors.

**Subject**	Sex	Age (years)	Weight (Kg)
**1**	W	29	55
**2**	W	25	44
**3**	W	25	42
**4**	W	22	60
**5**	W	21	64
**6**	W	42	66
**7**	W	29	64
**8**	M	26	93
**9**	M	21	102
**10**	M	24	75
**11**	M	36	105
**12**	W	22	51
**13**	M	24	74
**14**	M	22	56
**15**	M	23	80
**16**	M	20	90
**17**	W	53	85
**18**	M	28	77
**19**	M	23	71
**20**	M	25	80
**21**	M	25	120
**22**	W	24	60
**23**	W	60	70
**24**	W	34	64

Abbreviations: W, woman and M, man.

Table S2. Hematological parameters of healthy subjects.

Subject	WBC (K/µL)	Lymphocytes (%)	Neutrophils (%)	Eosinophils (%)	Basophils (%)	Mononuclear (%)	Platelets (K/µL)
**1**	9.09	44.4	43.9	4.42	1.36	5.88	287
**2**	6.58	33.6	58.1	0.5	1.12	6.7	177
**3**	6.6	27.1	63.3	1.03	0.91	7.59	194
**4**	5.9	45.2	45.9	1.07	0.95	6.88	188
**5**	8.37	48.6	43.5	1.65	0.76	5.43	232
**6**	6.85	33.9	57	3.03	1.04	5.06	176
**7**	4.63	27.4	59.8	2.61	0.78	9.36	160
**8**	6.9	39.7	50.3	3.45	2.21	4.38	127
**9**	7.33	33.1	54.1	3.82	1.33	7.65	183
**10**	6.88	43.8	43.8	3.17	1.27	7.9	174
**11**	5.61	39.6	50.7	2.06	1.94	5.76	191
**12**	6.85	23.7	69	0.8	1.24	5.3	189
**13**	6.09	40.9	46.2	2.74	1.38	8.78	247
**14**	5.05	39.7	48.9	2.13	1.63	7.69	237
**15**	7.71	25	65.2	3.68	0.6	5.47	246
**16**	6.45	22.4	65.5	2.49	1.04	8.55	165
**17**	10	28.4	59.7	2.57	1.77	7.53	286
**18**	6.6	41.7	47.2	3.72	1.42	5.96	177
**19**	5.33	39.4	52.9	0.86	1.38	5.49	245
**20**	7.67	38.3	51.1	1.02	1.7	7.94	193
**21**	nd	Nd	nd	nd	nd	nd	nd
**22**	9.13	35.9	56.1	2.01	1.1	4.9	246
**23**	5.84	37	53.5	3.3	1.3	4.9	273

Abbreviations: WBC, white blood cell counts (K/µL or absolute cell count/µL). nd – not done
